# Characterizing Music Engagement and Reward in Individuals Across a Spectrum of Hearing Loss and Rehabilitation

**DOI:** 10.1002/ohn.70340

**Published:** 2026-07-16

**Authors:** Emily Gao, Maria Armache, Joon Soo Kim, Celine Arar, Elaine C. Thompson, Claire Wang, Marshall Chasin, Theodore R. McRackan, Alexander Chern

**Affiliations:** ^1^ Department of Otolaryngology–Head and Neck Surgery Johns Hopkins University School of Medicine Baltimore Maryland USA; ^2^ Johns Hopkins University School of Nursing Baltimore Maryland USA; ^3^ School of Communication Disorders and Sciences Western University London Ontario Canada; ^4^ Musicians' Clinics of Canada Toronto Ontario Canada; ^5^ Department of Otolaryngology–Head and Neck Surgery Medical University of South Carolina Charleston South Carolina USA; ^6^ Department of Otorhinolaryngology–Head and Neck Surgery Hospital of the University of Pennsylvania Philadelphia Pennsylvania USA

**Keywords:** aural rehabilitation, cochlear implant, hearing loss, hearing aids, music perception, music engagement, music reward, music appreciation, quality of life

## Abstract

**Objective:**

Hearing aids (HAs) and cochlear implants (CIs) improve speech perception for individuals with hearing loss (HL), yet their impact on music outcomes remains unclear. We examined music perception, engagement, and reward in a large cohort of individuals across various hearing conditions.

**Study design:**

Cross‐sectional survey.

**Setting:**

Tertiary academic medical center and community.

**Methods:**

Adults (≥18 years) including normal hearing (NH) individuals, unaided individuals with HL, HA users, and CI users were assessed using the Barcelona Music Reward Questionnaire (BMRQ), Goldsmiths Musical Sophistication Index perceptual abilities subscale (GMSI‐PA), and music engagement items. ANOVA/ANCOVA was used to assess group differences, adjusting for age, gender, race, education, and musical training.

**Results:**

Among 201 participants (NH = 30, unaided HL = 23, HA = 84, CI = 64; 55.2% female; mean age 61.3), hearing status was associated with perceptual abilities; in particular, CI users had lower perceptual abilities than other groups (CI adjusted mean = 33.1 vs 38.6‐42.9 for NH, unaided HL, HA; all *P* < .01). However, non‐perceptual factors, including musical training and age, were stronger determinants of engagement and reward than hearing condition. A 1‐SD increase in musical training (10.1 points) was associated with a 3.1‐point increase in GMSI‐PA, 21.7 additional listening days/year, 32.3 additional participation days/year, and 3.1‐point increase in BMRQ.

**Conclusion:**

Poorer hearing status was associated with reduced music perception but not engagement or reward, even among CI users. Non‐perceptual factors like musical training and age may play a stronger role in explaining individual differences, highlighting the role of modifiable behavioral factors in enhancing music reward for individuals with HL.

Hearing loss (HL) is highly prevalent, significantly undertreated, and associated with a range of adverse health and quality of life (QOL) outcomes.^1^, ^2^, ^3^, ^4^, ^5^, ^6^, ^7^, ^8^, ^9^ Additionally, HL‐associated functional difficulties, particularly in complex listening tasks such as speech‐in‐noise and music perception, may hinder music engagement (ie, participation in a musical activities ranging from passive listening to active participation in a musical activity such as singing, playing an instrument, or dancing).^10^, ^11^, ^12^

Hearing devices (HDs) such as hearing aids (HAs) and cochlear implants (CIs) are primary tools for auditory rehabilitation and have led to significant improvements in speech perception.^13^, ^14^, ^15^ However, significant challenges remain in perceiving complex auditory signals, such as speech‐in‐noise and music.^16^, ^17^, ^18^, ^19^

Key components of the musical experience beyond music engagement include music perception and reward.^20^ Music perception is the ability to process and interpret elements of music such as pitch, rhythm, timbre, and melody, while music reward is the pleasurable, emotional, and overall sense of fulfillment associated with music.^10^, ^20^, ^21^, ^22^ Prior research has demonstrated lower perceptual abilities (eg, pitch and timbre perception) and reward in CI users.^21^, ^22^ Yet, far less is known about musical experiences in HA users and unaided individuals with HL, despite these groups representing the majority of the HL population.^23^ Research on HA users has shown that HAs may improve music reward for those with moderate to moderately severe HL.^10^, ^11^ However, research examining music engagement and reward across the full spectrum of hearing remains limited.

Our study aims addresses this gap by examining music engagement and reward in a large cohort of individuals spanning a range of hearing conditions—normal hearing (NH), unaided HL, HA users, and CI users. We hypothesized that more severe hearing conditions are associated with decreased music perception (eg, pitch, timbre) and engagement but may not correlate with broader, more subjective measures, such as music reward.

## Methods

### Participants

We recruited a convenience sample of demographically diverse participants with a range of music preferences and backgrounds across a spectrum of hearing conditions (NH, unaided HL, HA, CI) in‐person from otolaryngology clinics and virtually through HL support groups from 11/2024 to 8/2025. Single‐sided deafness, asymmetric SNHL (≥15 decibel hearing level [dB HL] pure tone average [PTA] difference between 2 ears), conductive HL, bone‐anchored HA users, non‐English‐speaking individuals, and those without internet access were excluded. The research protocol was approved by the Johns Hopkins Institutional Review Board IRB004112006.

### Protocol

Participants completed a Research Electronic Data Capture (REDCap) survey focused on demographics, music reward and sophistication questionnaires (ie, Barcelona Music Reward Questionnaire [BMRQ], Goldsmiths Musical Sophistication Index [GMSI]), music engagement items (eg, listening, active engagement), and hearing‐related items (eg, HA use).^24^, ^25^ Unaided 4‐frequency PTA (0.5, 1, 2, and 4 kHz) of the better hearing ear (PTAB) for non‐CI users and aided speech recognition scores (either Consonant‐Nucleus‐Consonant word or AZbio in quiet) for CI users were collected from participants' most recent hearing test results.

### Quantitative Analysis

We examined associations between hearing cognition and music perception, engagement, and reward.

#### Exposure Variable

Our exposure variable was the hearing condition, categorized as NH, unaided HL, HA use, and CI use.

#### Outcome Variables

Our primary outcomes of interest were music perception, engagement, and reward.

Music perception and musical training were measured using the GMSI perceptual abilities subscale (GMSI‐PA) and musical training subscale (GMSI‐MT, considered a covariate), components of a self‐report inventory assessing individual differences in innate musicality or musical sophistication (ie, ability to engage with music in a flexible, effective, and comprehensive way).^25^ Higher GMSI‐PA scores (range 9‐63) indicate increased accuracy of listening skills (eg, identifying when others are singing or playing out of tune).^25^ Higher GMSI‐MT scores (range 7‐49) indicate increased formal musical training received (eg, number of instruments played, including voice, years of study, hours of daily practice).^25^

Music engagement was defined as annual and daily frequency of music listening and annual participation in musical activities (eg, playing music, singing, or dancing).^26^, ^27^ The three items were: “How often do you listen to music currently?” “How often do you take part in musical activities (eg, choirs, orchestras, musicals or bands, play an instrument, sing, or dance)?” with answers ranging from “Once a year or less” to “Every day” as well as: “I listen attentively to music for __ per day,” from the GMSI with answers ranging from 1: “0–15 minutes” to 7: “4 hours or more.” To analyze our annual frequency results, we mapped the ordinal values to estimated days per year to make the variables continuous: 1 (0.5 days [once a year or less]), 2 (4 days [several times a year]), 3 (48 days [several times a month]), 4 (182 days [several times a week]), 5 (365 days [every day]).

Music reward was measured using the Barcelona Music Reward Questionnaire (BMRQ).^24^ Overall scores from this 20‐item validated test battery can range from 20 to 100, with higher scores indicating greater pleasure from engaging in any and all musical experiences.^24^

#### Covariates

Covariates included in multivariable models were age (years), sex (man/woman), education level (ranging from “less than high school degree” to “doctoral degree or professional degree”), race, and musical training (GMSI‐MT).

#### Statistical Analysis

Descriptive statistics were provided for continuous (mean, standard deviation) and categorical (frequency, proportion) variables. One‐way analysis of variance (ANOVA) assessed associations between hearing condition and outcome variables, followed by one‐way analysis of covariance (ANCOVA) adjusting for age, sex, education, race, HA use, and GMSI‐MT. For one‐way ANOVA models, we estimated unadjusted group means and two‐sided 95% confidence intervals (95% CI) from the fitted model and conducted post‐hoc pairwise comparisons. Similarly, for one‐way ANCOVA models, we estimated least‐squares (adjusted) means with 95% CI. Post‐hoc pairwise comparisons with Holm correction were performed to adjust for multiple comparisons.

Data analysis was performed using Python (Anaconda and Jupyter Notebook). *P*‐values were considered statistically significant at the *P* < .05 level (two‐tailed).

### Reporting

We followed the Strengthening the Reporting of Observational Studies in Epidemiology (STROBE) Cross‐Sectional Guidelines.

## Results

### Demographics and Descriptive Statistics

A total of 201 participants (55.2% female; mean age 61.3 years [range 19‐89]) met the inclusion criteria, including 30 NH individuals, 23 individuals with unaided HL, 84 HA users, and 64 CI users. Among non‐CI users (N = 137), the average participant had mild SNHL with a PTAB of 40.0 dB HL (range −10.0–105.0 dB HL). Mean PTAB differed by hearing group, with NH participants averaging 10.6 dB HL (SD 7.3), unaided HL participants 24.2 dB HL (SD 12.33), and HA users 53.9 dB HL (SD 19.6). Unaided HL participants most commonly exhibited a downsloping high‐frequency mild sensorineural HL, accounting for the group's average PTAB falling within the clinically normal range (≤25 dB HL). Among CI users, the average CNC word recognition score of the better ear among the 64 CI users was 63.9% (range 0%‐98%); 33 were bilateral CI users, 21 were contralateral HA users, and 10 were contralaterally unaided deafened individuals.

Across the entire cohort, average music listening was approximately every other day (224.2 days/year, range 0.5‐365) and an hour daily (57.8 minutes/day, range 7.5‐240), and music participation was lower at approximately 1 day per week (74.2 days/year, range 0.5‐365). The mean music perception score (GMSI‐PA) was 39.0 (range 9‐63 of a possible 9‐63), corresponding to approximately the 8th percentile of the original normative study with NH individuals.^25^, ^28^ Music training levels (GMSI‐MT) were more comparable to normative values, with a mean score of 21.0 (range 7‐45 of a possible 7‐49), corresponding to approximately 37th percentile of the original study.^25^, ^28^ Mean music reward (BMRQ total) was 72.7 (range 20‐100 of a possible 20‐100), which was slightly lower than the mean (78.4) of the original normative study with NH individuals.^24^ Further demographics and descriptive statistics are described in Table 1.

**Table 1:** Demographics

Characteristic	N = 201
Age, mean years (SD)	61.3 (18.8)
Sex, n (%)	
Male	90 (44.8)
Female	111 (55.2)
Race, n (%)	
White	167 (83.0)
Black or African American	20 (10.0)
American Indian or Alaska Native	0 (0.0)
Asian	7 (3.5)
Native Hawaiian or Pacific Islander	1 (0.5)
Other	5 (2.5)
Prefer not to respond	1 (0.5)
Education, n (%)	
Less than high school degree	1 (0.5)
High school graduate	8 (4.0)
Some college but no degree	18 (9.0)
Associate degree in college	13 (6.5)
Bachelor's degree in college	60 (29.9)
Master's degree	56 (27.9)
Doctoral or professional degree	45 (22.4)
Hearing demographics	
Non‐CI users, n (%)	137 (68.2)
Normal hearing individuals, n (%)	30 (14.9)
Individuals with unaided hearing loss, n (%)	23 (11.4)
HA users, n (%)	84 (41.8)
Pure tone average of better ear, mean dB HL (SD)	40.0 (24.8)
Normal, PTAB < = 25 dB HL, n (%)	47 (23.4)
Mild, PTAB 25.1‐40 dB HL, n (%)	24 (11.9)
Moderate, PTAB 40.1‐70 dB HL, n (%)	45 (22.4)
Severe to profound, PTAB > 70 dB HL, n (%)	17 (8.5)
CI users, n (%)	64 (31.8)
Bilateral CI users, n (%)	33 (51.6)
CI users with HA in contralateral ear, n (%)	21 (32.8)
CI user with unaided contralateral ear, n (%)	10 (15.6)
Consonant‐Nucleus‐Consonant score of (better hearing) CI ear, mean % correct (SD)	63.9 (26.2)
Pure tone average of non‐implanted ear, mean dB (SD) HL	79.8 (23.3)
Music demographics	
Musical training, mean GMSI musical training (SD)	21.0 (10.1)
Music perception, mean GMSI perceptual abilities (SD)	39.0 (11.1)
Music engagement frequency	
Annual listening, mean days per year (SD)	224.2 (140.3)
Daily listening, mean minutes per day (SD)	57.8 (64.9)
Annual active music engagement, mean days per year (SD)	74.2 (116.8)
Music reward, mean BMRQ (SD)	72.7 (14.1)
Musical sophistication, mean GMS (SD)	63.5 (19.7)

Abbreviations: BMRQ, Barcelona Music Reward Questionnaire; CI, cochlear implant; dB HL, decibel hearing level; GMS, General Musical Sophistication; GMSI, Goldsmiths Musical Sophistication Index; HA, hearing aid; SD, standard deviation.

### ANOVA and ANCOVA Results

Unadjusted analyses (ANOVA) demonstrated that hearing condition was associated with music perception, listening frequency, and music reward; however, after adjustment for age, sex, race, education, and musical training, hearing condition remained independently associated only with music perception. Adjustment‐attenuated associations with engagement and reward suggest that these outcomes were more strongly influenced by non‐perceptual factors. Because unadjusted and adjusted analyses showed consistent directional trends without qualitative discrepancies, only adjusted results are presented below. Primary outcome results are described below and in 2, 3, 4, 5 and 1, 2, 3.

**Table 2:** Unadjusted and Adjusted Model Associations of Hearing Condition With Perceptual Abilities, Music Engagement, and Music Reward

	Unadjusted (ANOVA)				Adjusted (ANCOVA)			
Outcomes	*F*(*df*)	*P*‐value	*η* ^2^	Effect size	*F*(*df*)	*P*‐value	Partial *η* ^2^	Effect size
Perceptual abilities (GMSI‐PA)								
GMSI perceptual abilities subscore	17.1 (3, 196)	<.001	0.21	Large	7.4 (3, 187)	<.001	0.11	Medium
Music engagement								
Annual listening frequency (days/year)	3.9 (3, 189)	.01	0.06	Medium	0.2 (3, 180)	.88	0.004	Null
Daily listening frequency (minutes/day)	4.5 (3, 196)	.004	0.06	Medium	1.6 (3, 187)	.19	0.03	Small
Annual active music participation (days/year)	0.4 (3, 190)	.73	0.007	Null	1.2 (3, 181)	.31	0.02	Small
Music reward (BMRQ)								
BMRQ	3.1 (3, 193)	.03	0.05	Small	1.0 (3, 184)	.40	0.02	Small

Abbreviations: ANCOVA, analysis of covariance; ANOVA, analysis of variance; BMRQ, Barcelona Music Reward Questionnaire.

**Table 3:** Unadjusted and Adjusted Means of Hearing Condition With Perceptual Abilities, Music Engagement, and Music Reward

	Unadjusted (ANOVA) mean (95% CI)				Adjusted (ANCOVA) mean (95% CI)			
Outcomes	NH	Unaided HL	HA	CI	NH	Unaided HL	HA	CI
Perceptual abilities (GMSI‐PA)								
GMSI perceptual abilities subscore	48.2 (44.6‐51.7)	42.0 (37.9‐46.0)	39.6 (37.4‐41.7)	33.0 (30.5‐35.4)	42.9 (38.5‐47.4)	40.9 (36.5‐45.3)	38.6 (36.1‐41.1)	33.1 (30.4‐35.8)
Music engagement								
Annual listening frequency (days/year)	306.8 (252.7‐361.0)	241.5 (182.4‐300.6)	209.2 (179.7‐238.8)	205.6 (171.4‐239.7)	237.6 (173.2‐302.0)	214.8 (152.2‐277.3)	219.5 (185.0‐254.1)	210.0 (172.8‐247.2)
Daily listening frequency (minutes/day)	96.0 (73.2‐118.8)	58.7 (32.7‐84.7)	52.0 (38.3‐65.6)	47.0 (31.3‐62.7)	76.0 (47.5‐104.6)	37.8 (9.3‐66.2)	56.5 (40.4‐72.5)	50.2 (32.8‐67.7)
Annual active music participation (days/year)	88.9 (42.6‐135.2)	59.4 (10.1‐108.8)	67.7 (42.5‐93.0)	82.3 (53.1‐111.4)	70.6 (12.9‐128.3)	63.0 (8.4‐117.6)	62.6 (31.6‐93.6)	98.8 (65.4‐132.2)
Music reward (BMRQ)								
BMRQ	77.8 (72.7‐82.9)	71.6 (66.0‐77.4)	74.0 (71.0‐77.1)	69.0 (65.5‐72.4)	73.2 (66.9‐79.4)	72.0 (65.7‐78.2)	75.9 (72.3‐79.4)	72.2 (68.4‐76.1)

Abbreviations: 95% CI, 95% confidence interval; ANCOVA, analysis of covariance; ANOVA, analysis of variance; BMRQ, Barcelona Music Reward Questionnaire; CI, cochlear implant; HA, hearing aids; HL, hearing loss; NH, normal hearing.

**Table 4:** Post‐hoc Holm‐Corrected Between‐Groups Comparison of Hearing Status with Perceptual Abilities, Music Engagement, and Music Reward

	Unadjusted (ANOVA)			Adjusted (ANCOVA)		
Between‐groups comparison	Change (%)	95% Confidence interval (not Holm‐corrected)	Holm‐corrected *P*‐value	Change (%)	95% Confidence interval (not Holm‐corrected)	Holm‐corrected *P*‐value
**Perceptual abilities (GMSI‐PA)**						
Cochlear implant users						
CI vs NH	–15.2 (–28.2)	–19.6 to –10.9	<.001*	–9.8 (–18.2)	–14.8 to –4.9	<.001*
CI vs Unaided HL	–9.0 (–16.7)	–13.8 to –4.2	<.001*	–7.8 (–14.4)	–12.6 to –3.0	.006*
CI vs HA	–6.6 (–12.2)	–9.9 to –3.3	<.001*	–5.5 (–10.1)	–8.7 to –2.3	.005*
Hearing aid users						
HA vs NH	–8.6 (–15.9)	–12.8 to −4.4	<.001*	–4.4 (–8.1)	–9.3 to 0.4	.22
HA vs Unaided HL	–2.4 (–4.4)	–7.0 to 2.2	.31	–2.4 (–4.4)	–7.1 to 2.4	.66
Unaided hearing loss						
Unaided vs NH	–6.2 (–11.5)	–11.6 to –0.8	.05	–2.1 (–3.9)	–7.6 to 3.5	.66
**Music engagement**						
*Annual listening frequency (days/year)*						
Cochlear implant users						
CI vs NH	–101.3	–165.3 to –37.2	.01*	–27.6	–97.7 to 42.5	1.00
CI vs Unaided HL	–36.0	–104.2 to 32.3	.90	–4.8	–72.3 to 62.4	1.00
CI vs HA	–3.6	–48.8 to 41.5	.90	–9.5	–53.8 to 34.7	1.00
Hearing aid users						
HA vs NH	–97.6	–159.3 to –35.9	.01*	–18.0	–87.7 to 51.6	1.00
HA vs Unaided HL	–32.3	–98.4 to 33.8	.90	4.8	–62.4 to 71.9	1.00
Unaided hearing loss						
Unaided HL vs NH	–65.3	−145.5 to 14.9	.44	–22.8	–102.0 to 56.4	1.00
*Daily listening frequency (minutes/day)*						
Cochlear implant users						
CI vs NH	–49.0	–76.7 to –21.3	.004*	–25.8	–57.7 to 6.1	.56
CI vs Unaided HL	–11.7	–42.1 to 18.7	1.00	12.5	–18.5 to 43.5	.87
CI vs HA	–4.9	–25.7 to 15.9	1.00	–6.2	–27.0 to 14.6	.87
Hearing aid users						
HA vs NH	–44.0	–70.6 to –17.5	.006*	–19.6	–50.8 to 11.7	.87
HA vs Unaided HL	–6.7	–36.1 to 22.6	1.00	18.7	–12.0 to 49.4	.87
Unaided hearing loss						
Unaided vs. NH	–37.3	–71.9 to –2.7	.14	–38.3	–74.3 to –2.3	.22
*Annual active music participation (days/year)*						
Cochlear implant users						
CI vs NH	–6.6	–61.4 to 48.1	1.00	28.2	–34.7 to 91.0	1.00
CI vs Unaided HL	22.9	–34.5 to 80.2	1.00	35.8	–23.6 to 95.3	1.00
CI vs HA	14.5	–24.1 to 53.1	1.00	36.2	–3.5 to 75.9	.44
Hearing aid users						
HA vs NH	–21.2	–73.9 to 31.6	1.00	–8.0	–70.5 to 54.5	1.00
HA vs Unaided HL	8.3	–47.1 to 63.8	1.00	–0.4	–59.6 to 58.9	1.00
Unaided hearing loss						
Unaided vs NH	–29.5	–97.2 to 38.2	1.00	–7.6	–78.0 to 62.7	1.00
**Music reward (BMRQ)**						
Cochlear implant users						
CI vs NH	–8.9 (–11.1)	–15.0 to –2.7	.03*	–0.9 (–1.1)	–7.9 to 6.1	1.00
CI vs Unaided HL	–2.7 (–3.4)	–9.4 to 3.9	.84	0.3 (0.4)	–6.6 to 7.1	1.00
CI vs HA	–5.1 (–6.3)	–9.7 to 0.5	.15	–3.6 (–4.5)	–8.2 to 0.9	.70
Hearing aid users						
HA vs NH	–3.8 (–4.8)	–9.7 to 2.1	.63	2.7 (3.4)	–4.2 to 9.6	1.00
HA vs Unaided HL	2.3 (2.9)	–4.1 to 8.8	.84	3.9 (4.9)	–2.9 to 10.7	1.00
Unaided hearing loss						
Unaided vs NH	–6.1 (–7.6)	–13.8 to 1.5	.46	–1.2 (–1.5)	–9.1 to 6.8	1.00

Abbreviations: BMRQ, Barcelona Music Reward Questionnaire; CI, cochlear implant; GMSI, Goldsmiths Musical Sophistication Index; HA, hearing aid; HL, hearing loss; NH, normal hearing.

* indicates statistical significance (*P* < .05).

**Table 5:** Covariate Associations on Music Perception, Music Engagement, and Music Reward: Musical Training, Age, Sex, and Education

	Adjusted (ANCOVA)					
Covariates	Effect (% change)	95% CI	*P*‐value	Δ*R* ^2^ (%)	*f* ^2^	Effect size
**Perceptual abilities (GMSI‐PA)**						
Musical training (Change per + 1‐SD [10.1 points])	3.1 (5.7)	1.7 to 4.5	<.001*	6.8	0.10	Small
Age (Change per 10‐year increase)	–1.1 (–2.0)	–2.0 to –0.1	.02*	1.8	0.03	Small
Sex (Female vs. Male)	–2.8 (–5.2)	–5.5 to –0.2	.04*	1.6	0.02	Small
Education (Change per additional year of education)	0.1 (0.2)	–0.4 to 0.7	.64	0.1	0.00	Null
**Music engagement**						
*Annual listening frequency (days/year)*						
Musical training (Change per + 1‐SD [10.1 points])	21.7	2.5 to 41.0	.03*	2.1	0.03	Small
Age (Change per 10‐year increase)	–20.2	–33.1 to –7.4	.002*	4.1	0.05	Small
Sex (Female vs. Male)	–9.1	–46.5 to 28.3	.63	0.1	0.00	Null
Education (Change per additional year of education)	–14.7	–22.8 to –6.5	<.001*	5.4	0.07	Small
*Daily listening frequency (minutes/day)*						
Musical training (Change per + 1‐SD [10.1 points])	3.8	–5.2 to 12.8	.41	0.3	0.00	Null
Age (Change per 10‐year increase)	–5.5	–11.5 to 0.6	.08	1.4	0.02	Small
Sex (Female vs. Male)	1.1	–16.2 to 18.3	.90	0.0	0.00	Null
Education (Change per additional year of education)	–6.6	–10.4 to –2.9	.001*	5.2	0.07	Small
*Annual active music participation (days/year)*						
Musical training (Change per + 1‐SD [10.1 points])	32.3	15.0 to 49.6	<.001*	6.7	0.08	Small
Age (Change per 10‐year increase)	–1.1	–12.6 to –10.4	.85	0.0	0.00	Null
Sex (Female vs. Male)	18.1	–15.4 to 51.6	.29	0.1	0.00	Null
Education (Change per additional year of education)	–1.1	–8.4 to 6.2	.78	0.0	0.00	Null
**Music reward (BMRQ)**						
Musical training (Change per + 1‐SD [10.1 points])	3.1 (3.9)	1.1 to 5.1	.002*	4.2	0.05	Small
Age (Change per 10‐year increase)	–1.7 (–2.1)	–3.0 to –0.4	.01*	2.8	0.04	Small
Sex (Female vs. Male)	3.2 (4.0)	–0.6 to 7.0	0.10	1.2	0.02	Small
Education (Change per additional year of education)	–0.6 (0.8)	–1.4 to 0.2	.17	0.1	0.01	Null

Abbreviations: BMRQ, Barcelona Music Reward Questionnaire; CI, confidence interval; GMSI, Goldsmiths Musical Sophistication Index; SD, standard deviation.

* indicates statistical significance (*P* < .05).

**Figure 1:**
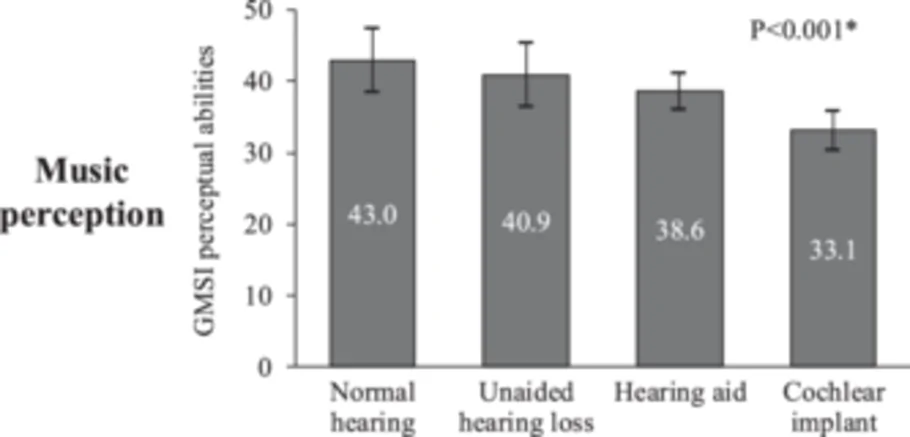
Adjusted least‐squares means ± 95% CI of hearing status and music perception. *Indicates statistical significance; CI, confidence interval.

**Figure 2:**
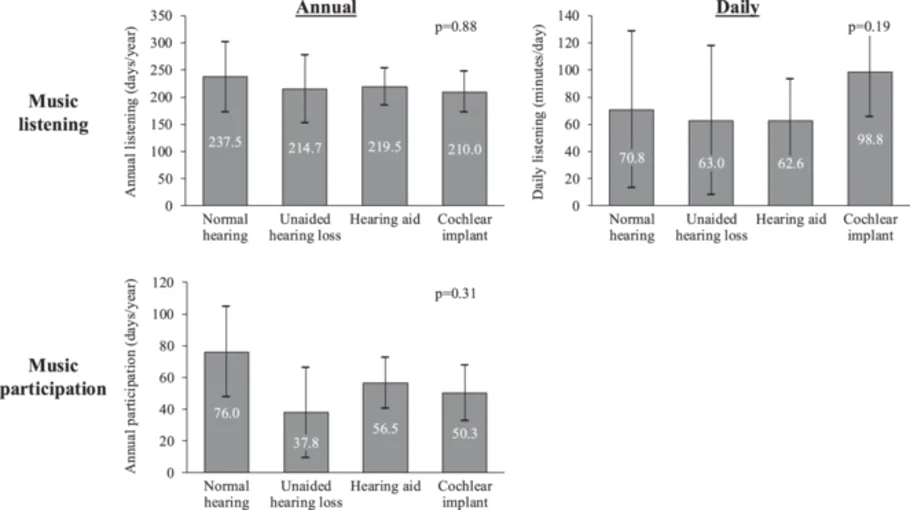
Adjusted least‐squares means ± 95% CI of hearing status and music engagement frequency. CI, confidence interval.

**Figure 3:**
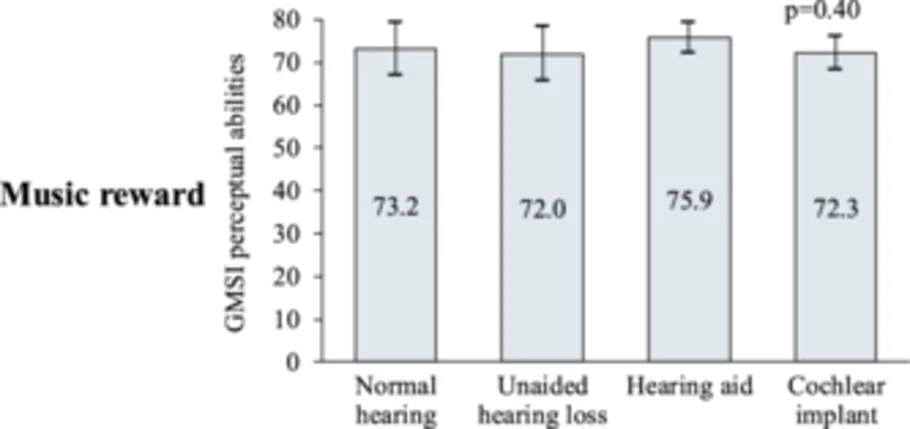
Adjusted least‐squares means ± 95% CI of hearing status and reward. CI, confidence interval.

### Music Perception (GMSI‐PA)

ANCOVA results indicated that hearing condition was moderately associated with GMSI‐PA (*P* < .001; *F*[3, 187] = 7.4; partial *η* ^2 ^= 0.11, medium effect), adjusting for covariates. Adjusted means demonstrated similar monotonic decreases (ie, progressively lower scores with increasing hearing impairment) across groups: NH (mean = 42.9, 95% CI; [38.5, 47.4]), unaided HL (mean = 40.9, 95% CI; [36.5, 45.3]), HA (mean = 38.6, 95% CI: [36.1, 41.1]), and CI (mean = 33.1, 95% CI; [30.4, 35.8]). Post‐hoc comparisons indicated that CI users exhibited lower perceptual abilities than all other groups, scoring 9.8 points (18.2%) below the NH group (95% CI: [4.9, 14.8]), *P* < .001), 7.8 points (14.4%) below the unaided HL group (95% CI: [3.0, 12.6], *P* = .006), and 5.5 points (10.1%) below the HA group (95% CI: [2.3, 8.7], *P* = .005).

Musical training, age, and sex were also significant predictors of musical perception. A 1‐SD (10.1 point) increase in musical training (GMSI‐MT) was associated with a small increase in perception (3.1 [5.7%] points; *P* < .001, 95% CI: [1.7, 4.5]; *f* ^2 ^= 0.10; small effect). Each 10‐year increase in age corresponded to a small decline in music perception (−1.1 [−2.0%] points; *P* = .02, 95% CI: [−2.0, −0.1]; *f* ^2 ^= 0.03, small effect). Finally, females reported slightly lower perception than males (−2.8 [−5.2%] points; *P* = .04, 95% CI: [−5.5, −0.2]; *f* ^2 ^= 0.02, small effect).

### Music Engagement (Listening and Active Participation Frequency)

ANCOVA results indicated the association between hearing condition and listening frequency was not significant, adjusting for covariates. Still, nonperceptual factors, including age and years of education, were predictors of music listening frequency, and musical training was associated with both music listening and participation. Each 10‐year increase in age corresponded to nearly 2 fewer days of music listening per month (−20.2 days/year; *P* = .002, 95% CI: [−33.1, −7.4]; *f* ^2 ^= 0.05, small effect). Every additional year of education was associated with small music listening decreases: approximately 1 day per month (−14.7 days/year; *P* < .001, 95% CI: [−22.8, −6.5]; *f* ^2 ^= 0.07, small effect) and 5 minutes per day (−6.6 minutes/day; *P* = .001, 95% CI: [−10.4, −2.9]; *f* ^2 ^= 0.07, small effect). Finally, a 1‐SD increase in musical training corresponding to nearly 2 additional listening (21.7 days/year; *P* = .03, 95% CI: [2.5, 41.0]; *f* ^2 ^= 0.03, small effect) and three additional participation days per month (32.3 days/year; *P* < .001, 95% CI: [15.0, 49.6]; *f* ^2^ = 0.08, small effect).

### Music Reward (BMRQ)

ANCOVA results demonstrated that the association between hearing condition and music reward was not significant, adjusting for covariates. There were minimal group differences in music reward: individuals with NH scored 73.2 points on the BMRQ (95% CI: [66.9, 79.4]), individuals with unaided HL scored 72.0 points (95% CI: [65.7, 78.2]), HA users scored 75.9 (95% CI: [72.3, 79.4]), and CI users scored 72.2 points (95% CI: [68.4, 76.1]).

Still, musical training and age were correlated with overall reward. A 1‐SD increase in GMSI‐MT was associated with a 3.1‐point increase in [3.9%] BMRQ; *P* = .002, 95% CI: [1.1, 5.1]; *f* ^2 ^= 0.05, small effect). Yet, each 10‐year increase in age corresponded to a small decrease in music reward (−1.7‐point [−2.1%] BMRQ; *P* = .01, 95% CI: [−3.0, −0.4]; *f* ^2 ^= 0.04, small effect).

## Discussion

Across the spectrum of hearing conditions, worsening hearing condition was strongly associated with decreased music perception, with CI users demonstrating the lowest perceptual scores even after adjusting for covariates. In contrast, hearing status was not associated with frequency of music engagement or reward after adjusting for covariates. Together, these findings suggest that hearing status may predict perceptual abilities but not how often individuals engage with music nor the reward they derive from it. Instead, musical training emerged as one of the strongest predictors across several outcomes, while age and education were consistent but weaker predictors of the musical experience. These results underscore that music perception, engagement, and reward represent distinct and partially independent domains, and even individuals with considerable hearing impairment, such as CI users, may maintain robust music reward despite perceptual limitations.

Our findings are consistent with prior work showing that worsening HL is associated with decreased music perception (eg, pitch and timbre discrimination).^10^, ^11^, ^29^, ^30^ Still, most existing studies have focused on 1 or 2 subgroups (eg, CI vs HA) and have emphasized music perception rather than broader experiential outcomes.^10^, ^11^, ^30^, ^31^, ^32^, ^33^, ^34^, ^35^, ^36^, ^37^, ^38^, ^39^ This present study expands the existing literature by evaluating not only perceptual outcomes but music reward and engagement in a well‐powered cohort of NH individuals, HA users, unaided individuals with HL, and CI users. Prior studies often assumed that reduced perceptual accuracy or sound quality directly implies reduced music reward.^10^, ^11^, ^22^, ^23^, ^29^ Our results challenge this assumption; while hearing status was associated with music perception, it did not predict music engagement or reward after adjusting for covariates.

Our findings align with the distinction between hearing health and auditory wellness proposed by Humes et al, which emphasizes that audiometric thresholds alone do not adequately capture the full experience of everyday listening, including comprehension of speech, perception of complex nonspeech sounds (eg, music, natural sounds), and localization.^40^ In our findings, musical training and age were noted to be more important drivers of music reward than to hearing status, and hearing condition may not fully predict music reward. Additionally, music reward may be amenable to rehabilitative strategies such as structured musical training, which has recently been explored as a rehabilitation tool for both children and adults with HL.^41^, ^42^, ^43^, ^44^, ^45^, ^46^ It may be a particularly effective strategy in older adults, particularly those at risk for cognitive decline, social isolation, and other neuropsychiatric disorders of aging.^45^, ^46^ Music engagement has been shown to improve cognition through greater cognitive reserve, preserved brain volume, and neural pathway activation for executive function, memory, language, and emotions,^47^, ^48^, ^49^, ^50^ as well as overall wellbeing and QOL by increasing emotional competence and prosocial behavior while reducing social isolation.^26^, ^51^, ^52^, ^53^, ^54^, ^55^ Thus, sustaining music engagement despite HL may support delaying or reducing cognitive decline and support overall wellbeing. Behavioral change in older adults can be challenging, but counselling from clinicians and support from younger family members may facilitate technology use and ultimately improve music engagement.^56^

Self‐management of HL among individuals with mild to moderate HL has expanded as new consumer hearing technologies (eg, integration of clinical‐grade HAs into consumer‐grade headphones) and over‐the‐counter HAs become more widespread, driving need for novel self‐management tools including ways to self‐assess auditory wellness.^40^ Modern HDs (including over‐the‐counter [OTC] devices) offer direct streaming and user‐adjustable auditory settings via smartphone apps. Additionally, consumer‐grade headphones are beginning to offer clinical‐grade HA software for those with mild to moderate SNHL, which may be an attractive option for HL individuals without HAs. While these innovations may be promising for addressing music‐specific concerns, technology adoption is still a known challenge for older adults, which may help explain the small association between age and music reward.

Our study had several limitations. Each hearing status (ie, NH, unaided HL, HA, CI) was composed of individuals with a heterogeneous range of hearing thresholds and speech recognition ability with varying musical experiences, which may explain why we did not detect statistically significant differences in some comparisons, particularly between NH individuals, unaided HL listeners, and HA users. Importantly, however, the effect‐size pattern consistently followed the expected gradient of auditory impairment, likely reflecting underlying differences in HL severity, with CI users showing the largest reductions in perceptual scores, followed by HA users, unaided HL listeners, and NH individuals. Such heterogeneity likely compressed mean differences within groups while preserving the overall directional trend. However, the focus of this paper was to address differences across each clinically relevant hearing condition. Although average PTAB among the unaided HL group fell within the clinically normal range (≤25 dB HL), this reflected predominantly downsloping mid‐ to high‐frequency mild sensorineural HL. Consistent with this profile, unaided HL participants demonstrated milder hearing impairment than HA users and were not using amplification at the time of assessment. HA fit could not be assessed because participants were recruited remotely through HL support groups rather than evaluated in person. Complete audiologic records were unavailable for a small minority (6%) of participants, particularly among individuals implanted more than 30 years ago who lacked recent testing. Furthermore, HL history, particularly age of diagnosis, duration of HL, and time with HDs, were not included in the analysis, which may have had downstream effects on early music exposure and prior musical training, particularly in older individuals with congenital or early‐onset HL who were unable to take advantage of recent technological advancements (eg, improved fidelity of sound, device streaming) early on in their disease progress.

Despite these challenges, hearing condition is a clinically relevant and easily understood stratification of the patient population, even more intuitive for patients and caregivers compared to audiometric thresholds which may require more nuanced clinical understanding. Trends in hearing health, such as the growing HL population both in younger (eg, due to increased noise insults such as loud concerts and in‐ear headphones) and older adult populations, expansion of the OTC HA market, and increasing attention on earlier phases of HL (eg, subclinical HL) underscore the need to empower patients with education and self‐management tools.^40^, ^57^, ^58^, ^59^, ^60^ Thus, stratifying by hearing condition may offer a more patient‐friendly framework.

Other limitations include participant self‐selection into the study, which may increase bias toward individuals with baseline interest in music, including individuals with HL who still enjoyed music or previously had enjoyed music but had significant current challenges with music engagement. Additionally, the BMRQ and GMSI have not been validated in the HL population. However, this limitation points toward the need for a validated construct or patient‐reported outcome measure assessing music reward that is informed by hearing and hearing‐related factors, which remains our overarching goal.

Our study demonstrates that individuals across spectrum of HL can find music rewarding and may guide clinicians to address modifiable music‐specific and technological factors with their patients. Musical training and improving technology adoption may be promising avenues to enhance music reward. We hope to develop and implement interventions addressing modifiable music‐specific and technological factors that can improve music reward and engagement in this population.

## Conclusions

Although poorer hearing status was linked to reduced music perception, musical training and age may play a stronger role in explaining individual differences. While perceptual abilities varied widely, differences in overall engagement or reward among hearing conditions were modest, suggesting factors beyond perception (eg, musical background, age) may play a larger role. Findings highlight the potential role of musical training and technology adoption in enhancing music reward for individuals with HL.

## Author Contributions

**Emily Gao**, conception and design, acquisition of data, analysis/interpretation of data, drafting/critically revising/finalizing the manuscript; **Maria Armache**, conception and design, drafting/critically revising the manuscript; **Joon Soo Kim**, acquisition of data, drafting/critically revising the manuscript; **Celine Arar**, design, acquisition of data, analysis/interpretation of data, drafting/critically revising the manuscript; **Elaine C. Thompson**, design, drafting/critically revising the manuscript; **Claire Wang**, design, drafting/critically revising the manuscript; **Marshall Chasin**, conception and design, drafting/critically revising the manuscript; **Theodore R. McRackan**, conception and design, analysis/interpretation of data, drafting/critically revising the manuscript; **Alexander Chern**, conception and design, acquisition of data, analysis/interpretation of data, critically revising/finalizing the manuscript.

## Disclosures

### Competing interests

None.

### Funding source

American Academy of Otolaryngology‐Head and Neck Surgery Health Services Research Grant, American Neurotology Society Research Grant.
